# Association of Drug Metabolic Enzyme Genetic Polymorphisms and Adverse Drug Reactions in Patients Receiving Rifapentine and Isoniazid Therapy for Latent Tuberculosis

**DOI:** 10.3390/ijerph17010210

**Published:** 2019-12-27

**Authors:** Ya-Yen Yu, Shih-Ming Tsao, Wen-Ta Yang, Wei-Chang Huang, Ching-Hsiung Lin, Wei-Wen Chen, Shun-Fa Yang, Hui-Ling Chiou, Yi-Wen Huang

**Affiliations:** 1 Institute of Medicine, Chung Shan Medical University, Taichung 402, Taiwan; yy68642@gmail.com (Y.-Y.Y.); ysf@csmu.edu.tw (S.-F.Y.); 2 Department of Clinical Laboratory, Changhua Hospital, Changhua 513, Taiwan; 3 Division of Chest, Department of Internal Medicine, Chung Shan Medical University Hospital, Taichung 402, Taiwan; tsmhwy@ms24.hinet.net; 4 Institute of Biochemistry, Microbiology and Immunology, Chung Shan Medical University, Taichung 402, Taiwan; 5 Department of Internal Medicine, Taichung Hospital, Ministry of Health and Welfare, Taichung 403, Taiwan; taic3057@gmail.com; 6 Division of Chest Medicine, Department of Internal Medicine, Taichung Veterans General Hospital, Taichung 407, Taiwan; huangweichangtw@gmail.com; 7 Department of Medical Technology, Jen-Teh Junior College of Medicine, Nursing and Management, Miaoli 356, Taiwan; 8 Department of Life Sciences, National Chung Hsing University, Taichung 402, Taiwan; 9 Department of Industrial Engineering and Enterprise Information, Tunghai University, Taichung 407, Taiwan; 10 Division of Chest, Changhua Christian Hospital, Changhua 500, Taiwan; 47822@cch.org.tw; 11 Department of Health, Pulmonary and Critical Care Unit, Changhua Hospital, Changhua 500, Taiwan; sweefa818@gmail.com; 12 Department of Medical Research, Chung Shan Medical University Hospital, Taichung 402, Taiwan; 13 School of Medical Laboratory and Biotechnology, Chung Shan Medical University, Taichung 402, Taiwan; 14 Department of Clinical Laboratory, Chung Shan Medical University Hospital, Taichung 402, Taiwan

**Keywords:** drug metabolic enzymes, polymorphism, latent tuberculosis, adverse drug reaction

## Abstract

Weekly rifapentine and isoniazid therapy (3HP) is the most frequent treatment for latent tuberculosis infection (LTBI). However, the association between major adverse drug reactions (ADRs) and drug metabolic enzyme single-nucleotide polymorphisms (SNPs) remains unclear. In this study, 377 participants who received the 3HP regimen were recruited and examined for genotyping of CYP5A6, CYP2B6, CYP2C19, CYP2E1, and NAT2 SNPs. In our study, 184 participants (48.4%) developed ADRs. Moreover, CYP2C19 rs4986893 (TT vs. CC+CT, odds ratio [OR] [95% CI]: 2.231 [1.015–4.906]), CYP2E1 rs2070676 (CC vs. CG+GG, OR [95% CI]: 1.563 [1.022–2.389]), and CYP2E1 rs2515641 (CC vs. CT+TT, OR [95% CI]: 1.903 [1.250–2.898]) were associated with ADR development. In conclusion, CYP2C19 and CYP2E1 SNPs may provide useful information regarding ADRs in LTBI patients receiving the 3HP regimen.

## 1. Introduction

Tuberculosis (TB) is still prevalent worldwide. Up to one-third of the world’s population is estimated to be infected with *Mycobacterium tuberculosis* [1]. Additionally, although infected individuals have no signs or symptoms of TB disease and are not infectious, they still have a risk of active TB infection and becoming infectious. In Taiwan, TB ranks the highest for the number of new cases and is the main cause of death annually from communicable diseases in Taiwan.

A comprehensive effort to control TB by implementing directly observed therapy (DOT), a short-course program, for all patients with TB has been implemented since 2006 in Taiwan to reduce the incidence to half, from 67.4 per 100,000 population to 33.7 per 100,000 population, by 2016. Although the incidence rate has declined, it gradually slowed down to 43.9 per 100,000 population by 2016, less than half of the target. Thus, intensified efforts to reduce TB morbidity and transmission by implementing more laboratory diagnoses, active-TB case identification, and latent TB infection (LTBI) treatment have been commenced since 2016.

Apart from the traditional LTBI treatment regimen, that is, isoniazid (INH) 300 mg daily for 9 months (9H), in 2011 the Centers for Disease Control and Prevention recommended a short-course combination regimen of once-weekly INH and rifapentine for 12 weeks (3HP) through DOT [2,3]. Compared with other regimens, the 3HP regimen has practical advantages, such as a shorter treatment period, higher completion rate, and cost effectiveness [4]. Typically, the hepatotoxicity risk during LTBI treatment is much lower in the 3HP regimen than in the 9H regimen [5]. Therefore, the 3HP regimen was initiated in Taiwan in 2013, as it is in one of the high prevalence areas for viral hepatitis. However, the incidence of flu-like syndrome, one of the side effects of the 3HP regimen, is much higher in Taiwan (8% vs. 2.2%) [6,7] than in other countries. The occurrence of flu-like syndrome is also the common reason for the noncompliance of patients with the 3HP regimen.

Flu-like syndrome is one of the adverse reactions of rifapentine. Rifapentine induces cytochrome P450 enzymes and can also accelerate the metabolism of certain drugs, such as birth control pills and antiretroviral drugs [8]. By contrast, INH is the inhibitor of cytochrome P450 enzymes [9,10]. Therefore, clinicians should pay more attention to drug–drug interactions. Another study reported that women, Caucasians, elderly individuals, and individuals with a low body mass index (BMI) have a higher risk of developing systemic drug reactions after the administration of the 3HP regimen; however, the mechanism underlying this increased risk remains unclear [6]. Therefore, in this study, eight polymorphisms of drug metabolic enzyme genes, namely *CYP5A6* rs28399433, *CYP2B6* rs8192709, *CYP2C19* rs4986893, *CYP2C19* rs12248560, *CYP2E1* rs2070676, *CYP2E1* rs2515641, *NAT2* rs1495741, and *NAT2* rs1799930, were examined to study their associations with susceptibility to adverse drug reactions (ADRs) in the 3HP regimen in patients with latent TB infection in Taiwan.

## 2. Materials and Methods

### 2.1. Study Design

This was a multicenter observational study including close contacts aged >12 years and selecting individuals not resistant to INH and rifampin between February 2017 and October 2018. Participants received 15 mg/kg of INH (maximum dose of 900 mg) and rifapentine (maximum dose of 900 mg). Basic information, namely age, sex, BMI, ethnicity, education, comorbidities, medications, occupation, and compliance with therapy, were recorded for each participant. Dose adherence was assessed by DOT, and ADR monitoring was based on patients’ self-report. ADRs were recorded after receiving the first dose each week until two weeks after the treatment. The onset time, duration, and severity of ADRs, such as flu-like syndrome and skin symptoms, were recorded. Severity was defined by the common toxicity criteria of the Cancer Therapy Evaluation Program. The association between the cause of incomplete treatment and single-nucleotide polymorphisms (SNPs) was analyzed on the basis of the aforementioned data. Written informed consent was obtained from each participant enrolled in this study. This study was approved by the Institutional Review Board of Chung Shan Medical University Hospital (CSMUH No: CS16131).

### 2.2. Sample Preparation and DNA Extraction

Peripheral blood specimens were collected from patients with LTBI for genomic DNA extraction. Whole blood samples were placed in ethylenediaminetetraacetic (EDTA)-containing tubes and were centrifuged at 3000 rpm for 10 min. Genomic DNA extraction was performed using QIAamp DNA blood mini kits (Qiagen, Valencia, CA, USA) as previously described [11]. Extracted DNA was dissolved in Tris-EDTA (TE) buffer and was applied as DNA template in the following process of polymerase chain reactions.

### 2.3. Drug Metabolic Enzyme SNP Genotyping

Assessment of allelic discrimination for *CYP5A6* rs28399433 (assay ID: C_30634332_10), *CYP2B6* rs8192709 (assay ID: C_2818162_20), *CYP2C19* rs4986893 (assay ID: C_27861809_10), *CYP2C19* rs12248560 (assay ID: C_469857_10), *CYP2E1* rs2070676 (assay ID: C_16026001_20), *CYP2E1* rs2515641 (assay ID: C_16026002_10), *NAT2* rs1495741 (assay IDs: C_8684110_10), and *NAT2* rs1799930 (assay IDs: C_1204091_10) SNPs were performed using the TaqMan assay with an ABI StepOne Software v2.3 Real-Time PCR System. The real-time PCR consisted of initial denaturation at 95 °C for 10 min, followed by 40 cycles at 95 °C for 15 s and finally 1 min at 60 °C. Generated data were collected and further evaluated using the SDS 7000 series software (Applied Biosystems, Foster City, CA, USA).

### 2.4. Statistical Analysis

Continuous variables were determined using Student’s *t*-test, and categorical variables were determined using the chi-squared test or Fisher’s exact test. The chi-squared test was also applied to determine the prevalence of all drug metabolic enzyme gene polymorphisms, such as CYP2E1 genotype, between the non-ADR and ADR group. Multiple unconditional logistic regression analyses were performed to estimate odds ratios and their 95% confidence intervals. Two-tailed *p* values < 0.05 were considered to be statistically significant.

## 3. Results

A total of 377 TB patients were recruited and administered the 3HP regimen. Of these 377 participants, 337 (89.3%) completed the treatment, 29 (7.7%) discontinued treatment owing to ADRs, 8 (2.1%) switched to the 9H regimen due to ADRs, and 3 (0.8%) transferred to another facility or refused further treatment (Table 1). In the total sample, the mean age was 45.7 years, 208 participants (55.2%) were women, 144 participants (38.2%) had comorbidities, and 184 (48.8%) developed ADRs (Table 2). ADRs occurred in 184 participants (48.4%), and 596 adverse drug events were reported. Most of the ADRs were Grade 1 (77.68%), followed by Grade 2 ADRs (20.63%). Grade 3 ADRs were observed most frequently in patients with flu-like symptoms (80%), one patient with urticaria, and one patient with hepatotoxicity.

The genotypes of CYP5A6, CYP2B6, CYP2C19, CYP2E1, and NAT2 SNPs in the non-ADR and ADR groups are shown in Table 3. In the non-ADR and ADR groups, the highest distribution frequencies of the drug metabolic enzyme genetic polymorphisms rs28399433, rs8192709, rs4986893, rs12248560, rs2070676, rs2515641, rs1495741, and rs1799930 were homozygous for AA, TT, GG, CC, CC, CC, GA, and GG, respectively. As shown in Table 3, patients with CYP2B6 polymorphic rs8192709 TC or TC+CC, CYP2E1 polymorphic rs2070676 CG or CG+GG, CYP2E1 polymorphic rs2515641 CT or CT+TT genotypes exhibited significantly (*p* < 0.05) higher frequencies, 2.355-fold (95% CI: 1.037–5.351), 2.231-fold (95% CI: 1.015–4.906), 1.594-fold (95% CI: 1.031–2.464), 1.563-fold (95% CI: 1.022–2.389), 1.850-fold (95% CI: 1.193–2.870), and 1.903-fold (95% CI: 1.250–2.898) respectively, of developing ADRs compared with their corresponding wild type (WT) homozygotes.

The allele frequencies of CYP5A6, CYP2B6, CYP2C19, CYP2E1, and NAT2 SNPs in patients in the non-ADR and ADR groups are shown in Table 4. Results indicated that patients with the CYP2E1 polymorphic rs2515641 T allele and NAT2 polymorphic rs1495741 A allele exhibited significantly (*p* < 0.05) higher frequencies, 1.704-fold (95% CI: 1.200–2.421) and 1.415-fold (95% CI: 1.062–1.885), respectively, of developing ADRs compared with their corresponding WT homozygotes (Table 4).

## 4. Discussion

In this study, we investigated the significance of the relationship between ADR in patients with LTBI receiving the 3HP regimen and several drug metabolic enzyme gene polymorphisms of *CYP5A6*, *CYP2B6*, *CYP2C19*, *CYP2E1*, and *NAT2* in the Taiwanese population. Results showed that almost half of the participants (184/377, 48.8%) experienced ADRs (98.3% were <Grade 2), and the most common presentation was flu-like symptoms, accounting for 80.2% of ADR episodes, which is much higher than that reported in a previous study [6]. Our study results indicated that the high incidence of flu-like symptoms is the major problem associated with the prescription of 3HP short-term treatment for LTBI cases. This is another aspect that should be further investigated.

INH is an inhibitor of cytochrome P450 [9,10]; by contrast, rifapentine is an inducer of cytochrome P450 [8,12], which may result in pharmacodynamic reduction in the treatment regimen. Our study showed that cytochrome P450 enzymes, such as CYP2B6 and CYP2E1, were associated with the incidence of ADR (*p* < 0.05). Some of these enzymes are involved in the metabolic pathway of INH, such as CYP2E1 and NAT2 [13,14], and some have direct or indirect effects on INH, such as CYP2B6 [15]. Based on our study results, the occurrence of side effects appeared to be more related to INH. Thus, the relationship between INH and flu-like symptoms is worth examining because the major side effects of the 3HP treatment are flu-like symptoms.

The 3HP regimen contained 3 times higher doses, especially for INH, when compared with other regimens. However, based on animal studies, high doses of INH have no greater efficacy than standard doses (300 mg) [16]. By contrast, significant differences in drug tolerance, ADRs, and safety were not observed between the high and normal doses of rifapentine [17,18,19,20]. Rifapentine acts as an inducer of many metabolic enzymes, especially in antiretroviral drugs; thus, interactions between INH and rifapentine have been evaluated in many studies [21,22]. In 2018, Brooks et al. indicated that serious drug side effects are associated with endogenous cytokines [23]. Therefore, we speculate that high doses of INH can induce side effects of rifapentine, coupled with the effects of certain genotypes, resulting in more drug side effects in many cases. However, this hypothesis must be explored and confirmed by conducting additional molecular biology, pharmacokinetics, and pharmacodynamics studies, and even animal experiments.

This study aimed to determine whether the side effects of LTBI 3HP treatment (INH and rifapentine) are associated with SNPs. Many studies have discussed the association between genotypes and drug side effects [24,25,26]; with the rise of pharmacogenetics and pharmacogenomics in recent years [27,28,29], in addition to the current focus on personalized medicine, the results of this study are expected to be personalized in TB.

Several genotypes (rs8192709 TC, rs2070676 CG, rs2515641 CT, and rs1495741 AA) showed an association with side effects to LTBI in our study. The results indicate that for patients with the rs8192709 TC genotype, the risk of side effects is 2.355 folds higher than that for patients with the TT genotype. Furthermore, patients with the rs2070676 CG genotype, rs2515641 CT genotype, and rs1495741 AA genotype had higher risks of side effects (*p* = 0.036, *p* = 0.006, and *p* = 0.018, respectively) than did patients with other genotypes (Table 3). Additionally, rs2515641 T and rs1495741 A allele carriers belong to a high-risk group for side effects (*p* = 0.003 and *p* = 0.018, respectively) (Table 4). Hiratsuka et al. have suggested that SNPs of NAT2 enzymes can predict the side effects of INH [30]. Similarly, Sotsuka et al. indicated that hepatotoxicity caused by INH is related to the genotype of its drug-metabolizing enzyme [31]. Additionally, the genotype of NAT2 is associated with failure or recurrence of TB treatment [21]. Most of the studies have investigated the relationship between INH and genotypes. By contrast, few studies have focused on the relationship between rifapentine and genotypes; this may be due to the metabolic pathway of rifapentine, which is still unclear, thus hindering further research on genetic polymorphisms.

## 5. Conclusions

In summary, we found that CYP2C19 rs4986893, CYP2E1 rs2070676, and CYP2E1 rs2515641 were associated with ADR development. Since CYP2C19 and CYP2E1 have been proven to be involved in the metabolic pathway of INH, their association with ADR development is a rational supposition. The results of this study can provide an improved strategy for identifying people with increased TB risk.

## Figures and Tables

**Table 1:** Characteristics and treatment outcomes of the enrolled participants.

(*n* = 377)	Treatment Completed	Interrupted Due to Side Effects	Switched to 9H Due to Side Effects	Other *
Number	337	29	8	3
%	89.4%	7.7%	2.1%	0.8%

* Transferred to other medical facility, refused further treatment.

**Table 2:** Clinical characteristics and laboratory data in 377 patients.

Variable	(*n* = 377)	Non-ADR (*n* = 193)	ADR (*n* = 184)
Age (year)			
≦35	96	53	43
36–55	169	88	81
56–64	83	37	46
≧65	29	15	14
Gender			
Male	169	101	68
Female	208	92	116
Body mass index (kg/m^2^)	24.9	25	24.7
AST (U/L)	25.11	24.95	25.32
ALT (U/L)	27.08	26.98	27.1
Comorbidity (*n* = 144)			
Hypertension	46	22	24
Diabetes mellitus	21	12	9
HBV infection	17	11	6
Heart disease	8	4	4
Thalassemias	5	1	4
Hyperlipidemia	4	2	2
Allergic rhinitis	4	4	0
Insomnia	3	0	3
Hyperthyroidism	3	2	1
Gastric ulcer	4	0	4
Hyperthyroidism	3	2	1
Hepatitis C	2	0	2
Gout	2	0	2
Lung cancer	2	0	2
Dementia	1	0	1
Breast cancer	2	1	1
Proctitis	1	0	1
Fatty liver	1	0	1
Hyperuricemia	1	1	0
Others	14	8	6

ADR: adverse drug reaction; AST: aspartate transaminase; ALT: alanine transaminase. HBV: Hepatitis B virus; Others: epilepsy, urticaria, cerebral neurasthenia, cerebral palsy, fibromyalgia, anemia, aplastic anemia, gall stone, hemochromatosis. Data are number.

**Table 3:** Single nucleotide polymorphism frequencies of five drug-metabolic genes in 377 patients.

Variable	ALL (*n* = 377)	Non-ADR (*n* = 193)	ADR (*n* = 184)	OR (95% C.I.)	*p* Value
CYP5A6 (rs28399433)					
A/A	235 (62.3%)	116 (60.1%)	119 (64.7%)	1.000 (reference)	
C/A	100 (26.5%)	54 (28.0%)	46 (25.0%)	0.830 (0.519–1.327)	*p* = 0.437
C/C	42 (11.2%)	23 (11.9%)	19 (10.3%)	0.805 (0.417–1.557)	*p* = 0.520
C/A+C/C	142 (37.7%)	77 (39.9%)	65 (35.3%)	0.823 (0.542–1.249)	*p* = 0.360
CYP2B6 (rs8192709)					
T/T	347 (92.0%)	183 (94.8%)	164 (89.1%)	1.000 (reference)	
T/C	28 (7.5%)	9 (4.7%)	19 (10.3%)	**2.355 (1.037–5.351)**	*p* = 0.041 *
C/C	2 (0.5%)	1 (0.5%)	1 (0.6%)	1.116 (0.069–17.983)	*p* = 0.938
T/C+C/C	30 (8.0%)	10 (5.2%)	20 (10.9%)	**2.231 (1.015–4.906)**	*p* = 0.046 *
CYP2C19 (rs4986893)					
G/G	342 (90.7%)	180 (93.3%)	162 (88.0%)	1.000 (reference)	
G/A	34 (9.0%)	12 (6.2%)	22 (12.0%)	2.037 (0.977–4.247)	*p* = 0.058
A/A	1 (0.3%)	1 (0.5%)	0 (0.0%)	-	-
G/A+A/A	35 (9.3%)	13 (6.7%)	22 (12.0%)	1.880 (0.917–3.854)	*p* = 0.085
CYP2C19 (rs12248560)					
C/C	375 (99.5%)	191 (99.0%)	184 (100.0%)	1.000 (reference)	
T/C	2 (0.5%)	2 (1.0%)	0 (0.0%)	-	-
T/T	0 (0%)	0 (0.0%)	0 (0.0%)	-	-
T/C+T/T	2 (0.5%)	2 (1.0%)	0 (0.0%)	-	-
CYP2E1 (rs2070676)					
C/C	243 (64.5%)	134 (69.4%)	109 (59.3%)	1.000 (reference)	
C/G	124 (32.9%)	54 (28.0%)	70 (38.0%)	**1.594 (1.031–2.464)**	*p* = 0.036 *
G/G	10 (2.6%)	5 (2.6%)	5 (2.7%)	1.229 (0.347–4.356)	*p* = 0.749
C/G+G/G	134 (35.5%)	59 (30.6%)	75 (40.8%)	**1.563 (1.022–2.389)**	*p* = 0.039 *
CYP2E1 (rs2515641)					
C/C	232 (61.5%)	133 (68.9%)	99 (53.8%)	1.000 (reference)	
C/T	126 (33.4%)	53 (27.5%)	73 (39.7%)	**1.850 (1.193–2.870)**	*p* = 0.006 *
T/T	19 (5.1%)	7 (3.6%)	12 (6.5%)	2.303 (0.875–6.062)	*p* = 0.091
C/T+T/T	145 (38.5%)	60 (31.1%)	85 (46.2%)	**1.903 (1.250–2.898)**	*p* = 0.003 *
NAT2 (rs1495741)					
G/G	116 (30.8%)	63 (32.6%)	53 (28.8%)	1.000 (reference)	
G/A	166 (44.0%)	94 (48.7%)	72 (39.1%)	0.910 (0.565–1.467)	*p* = 0.700
A/A	95 (25.2%)	36 (18.7%)	59 (32.1%)	**1.948 (1.121–3.385)**	*p* = 0.018 *
G/A+A/A	261 (69.2%)	130 (67.4%)	131 (71.2%)	1.198 (0.773–1.857)	*p* = 0.420
NAT2 (rs1799930)					
G/G	219 (58.1%)	112 (58.0%)	107 (58.1%)	1.000 (reference)	
G/A	128 (33.9%)	62 (32.1%)	66 (35.9%)	1.114 (0.720–1.724)	*p* = 0.627
A/A	30 (8.0%)	19 (9.9%)	11 (6.0%)	0.606 (0.275–1.333)	*p* = 0.213
G/A+A/A	158 (41.9%)	81 (42.0%)	77 (41.8%)	0.995 (0.661–1.498)	*p* = 0.981

Note: * and bold text indicate a significant association with *p*-value < 0.05.

**Table 4:** Single nucleotide polymorphism frequencies of five drug-metabolic genes in 377 patients.

Variable	ALL (*n* = 754)	Non-ADR (*n* = 386)	ADR (*n* = 368)	OR (95% C.I.)	*p* Value
CYP5A6 (rs28399433)					
A allele	570 (75.6%)	286 (74.1%)	284 (77.2%)	1.000 (reference)	
C allele	184 (24.4%)	100 (25.9%)	84 (22.8%)	0.846 (0.606–1.181)	*p* = 0.325
CYP2B6 (rs8192709)					
T allele	722 (95.8%)	375 (97.2%)	347 (94.3%)	1.000 (reference)	
C allele	32 (4.2%)	11 (2.8%)	21 (5.7%)	2.063 (0.980–4.341)	*p* = 0.056
CYP2C19 (rs4986893)					
G allele	718 (95.2%)	372 (96.4%)	346 (94.0%)	1.000 (reference)	
A allele	36 (4.8%)	14 (3.6%)	22 (6.0%)	1.690 (0.851–3.355)	*p* = 0.134
CYP2C19 (rs12248560)					
C allele	752 (99.7%)	384 (99.5%)	368 (100.0%)	1.000 (reference)	
T allele	2 (0.3%)	2 (0.5%)	0 (0.0%)	-	-
CYP2E1 (rs2070676)					
C allele	610 (80.9%)	322 (83.4%)	288 (78.3%)	1.000 (reference)	
G allele	144 (19.1%)	64 (16.6%)	80 (21.7%)	1.398 (0.970–2.013)	*p* = 0.072
CYP2E1 (rs2515641)					
C allele	590 (78.3%)	319 (82.6%)	271 (73.6%)	1.000 (reference)	
T allele	164 (21.7%)	67 (17.4%)	97 (26.4%)	**1.704 (1.200–2.421)**	*p* = 0.003 *
NAT2 (rs1495741)					
G allele	398 (52.8%)	220 (57.0%)	178 (48.4%)	1.000 (reference)	
A allele	356 (47.2%)	166 (43.0%)	190 (51.6%)	**1.415 (1.062–1.885)**	*p* = 0.018 *
NAT2 (rs1799930)					
G allele	566 (75.1%)	286 (74.1%)	280 (76.1%)	1.000 (reference)	
A allele	188 (24.9%)	100 (25.9%)	88 (23.9%)	0.899 (0.646–1.251)	*p* = 0.528

Note: * and bold text indicated a significant association with *p*-value < 0.05.
